# Identifying Neural Patterns of Functional Dyspepsia Using Multivariate Pattern Analysis: A Resting-State fMRI Study

**DOI:** 10.1371/journal.pone.0068205

**Published:** 2013-07-12

**Authors:** Peng Liu, Wei Qin, Jingjing Wang, Fang Zeng, Guangyu Zhou, Haixia Wen, Karen M. von Deneen, Fanrong Liang, Qiyong Gong, Jie Tian

**Affiliations:** 1 Life Science Research Center, School of Life Science and Technology, Xidian University, Xi’an, People’s Republic of China; 2 Acupuncture and Tuina School, Chengdu University of Traditional Chinese Medicine, Chengdu, People’s Republic of China; 3 Institute of Automation, Chinese Academy of Sciences, Beijing, People’s Republic of China; 4 Department of Radiology, The Center for Medical Imaging, Huaxi MR Research Center, West China Hospital of Sichuan University, Sichuan, China; Hangzhou Normal University, China

## Abstract

**Background:**

Previous imaging studies on functional dyspepsia (FD) have focused on abnormal brain functions during special tasks, while few studies concentrated on the resting-state abnormalities of FD patients, which might be potentially valuable to provide us with direct information about the neural basis of FD. The main purpose of the current study was thereby to characterize the distinct patterns of resting-state function between FD patients and healthy controls (HCs).

**Methodology/Principal Findings:**

Thirty FD patients and thirty HCs were enrolled and experienced 5-mintue resting-state scanning. Based on the support vector machine (SVM), we applied multivariate pattern analysis (MVPA) to investigate the differences of resting-state function mapped by regional homogeneity (ReHo). A classifier was designed by using the principal component analysis and the linear SVM. Permutation test was then employed to identify the significant contribution to the final discrimination. The results displayed that the mean classifier accuracy was 86.67%, and highly discriminative brain regions mainly included the prefrontal cortex (PFC), orbitofrontal cortex (OFC), supplementary motor area (SMA), temporal pole (TP), insula, anterior/middle cingulate cortex (ACC/MCC), thalamus, hippocampus (HIPP)/parahippocamus (ParaHIPP) and cerebellum. Correlation analysis revealed significant correlations between ReHo values in certain regions of interest (ROI) and the FD symptom severity and/or duration, including the positive correlations between the dmPFC, pACC and the symptom severity; whereas, the positive correlations between the MCC, OFC, insula, TP and FD duration.

**Conclusions:**

These findings indicated that significantly distinct patterns existed between FD patients and HCs during the resting-state, which could expand our understanding of the neural basis of FD. Meanwhile, our results possibly showed potential feasibility of functional magnetic resonance imaging diagnostic assay for FD.

## Introduction

Functional dyspepsia (FD), as one of the most common clinical functional gastrointestinal diseases, is defined as ‘presence of symptoms thought to originate in the gastroduodenal region, in the absence of any organic, systemic, or metabolic disease likely to explain the symptoms’ [1]. Although FD has a profound impact on quality of the life of patients and yields quite high medical costs, the underlying pathogenesis of FD remains unclear. Fortunately, neuroimaging techniques, such as magnetic resonance imaging (MRI) and positron emission tomography (PET), provide us a window to investigate the dysfunction of the brain-gut axis involved in processing of visceral discomfort or pain, which has been suggested to play a critical role in FD disorder [2], [3], [4], [5], [6], [7].

Recently, functional MRI (fMRI) and PET have been largely used to rule out alternative causes of FD while measuring the patterns of brain activity, as the biological marker. Furthermore, various abnormalities of the brain regions in FD patients have been found in comparison with healthy controls (HCs). By applying experimental design of gastric balloon distention, Vandenberghe et al investigated that there existed abnormal activity in the brain regions between FD patients with hypersensitivity, consisting of the bilateral gyrus precentralis, bilateral gyrus frontalis inferior, bilateral gyrus frontalis medialis, bilateral gyrus temporalis superior, bilateral cerebellar hemisphere, and left gyrus temporalis inferior [7]. Van Oudenhove et al investigated the abnormal brain processing during the gastric distention stimulation, sham and no distension conditions. Abnormal brain activations, found between FD patients and HCs during different conditions, were associated with sensory, arousal-anxiety and affective-cognitive modulations [2].

In the other hand, the resting-state abnormalities of FD patients have been being paid more attention to, which might be potential valuable to provide us with more direct information about the neural basis of FD. Van Oudenhove and colleagues detected that psychosocial factors might contribute to FD abnormal brain activity during the resting-state. They reported that activity in the pregenual anterior cingulate cortex (pACC) and anterior middle cingulated cortex (aMCC) was negatively correlated with anxiety, while the dorsal pons activity was positively related with anxiety [2]. With regards to abuse history in FD, Van Oudenhove et al detected that abuse history was related to distinct activities in the insula, prefrontal cortex (PFC), hippocampus (HIPP) and amygdale [6]. Evidence from one of our previous studies suggested that there was an extensive increasing cerebral glycometabolism especially in the homeostatic afferent processing network, such as the ACC, MCC, insula and thalamus [3]. We also tried to investigate dysfunctional connectivity between brain regions between the cerebral hemisphere, and reported increased interhemispheric connectivity in brain regions including the ACC, insula and thalamus [8]. However, the number of FD-related resting-state studies still remained limited.

Although these findings mentioned above revealed the dysfunctions of the brain processing visceral information in FD patients, these studies mainly focused on determining statistical group differences by conventional methods, which analyzed activation of each position of the brain, separately. Although conventional methods have the ability of detecting differential neuronal processes, they ignore potential sources information or the spatial correlation in the data, such as the interrelationship between the activities at different brain areas [9], and may not be optimally suited to investigate the diagnostic separation between patients and HCs, based on neuroimaging data [10]. In recent years, pattern recognition techniques have been widely implicated in fMRI data analysis, referred to multivariate pattern analysis (MVPA). Since MVPA can take into account the pattern of information which may be shown across multiple variables, MVPA presents a higher sensitivity compared to the traditional statistical methods [11], [12]. This method also considers the conjoint information of different patterns. Therefore, it is widely used to detect the clinical diagnostic states [10], [13], [14], [15], [16], [17], [18]. Nevertheless, up until to now there was no study considering for analyzing abnormal brain activity of FD patients during the resting state, using the MVPA approach.

In the present study, our subjective was (I) to investigate whether there was a characteristic distribution pattern of brain activity that was different in the FD patients from the HCs during the resting-state. (II) to detect the interactions between the characteristic brain activity of the FD patients from the neuroimaging findings and the FD symptoms (i.e. disease duration and symptom severity). To achieve these goals, we firstly used support vector machines (SVM) as a pattern classifier, and we trained this classifier with a patient group of thirty individuals and a control group of thirty individuals using leave-one-out cross validation (LOOCV). A correlation analysis was then conducted for the relationship between the abnormal neuroimaging findings and FD symptoms. Here, we hypothesized that there existed certain characteristic distributed pattern that contributed to finding the FD-related abnormalities of brain activity during the resting-state, compared with HCs. We also hypothesized that the FD symptoms would have an effect on the characteristic distributed pattern of brain activity in FD patients.

## Materials and Methods

### Ethics Statement

All research procedures of the current study were approved by the West China Hospital Subcommittee on Human Studies and were conducted in accordance with the Declaration of Helsinki. Verbal and written consent was obtained from each subject.

### FD Patients

Thirty FD patients were identified according to Rome III criteria and were recruited in the present study. Inclusion criteria were: 1) aged between 20 and 30 years and 2) meeting the Rome III criteria on FD and postprandial distress syndrome (PDS). The patient was excluded if he/she met any of the following criteria: 1) being pregnant or lactating, 2) having taken medications such as selective serotonin reuptake inhibitors, non-steroidal anti-inflammatory drugs, aspirin, phenothiazines and steroids that affect gastrointestinal motility for over two weeks prior to enrollment or having other substance abuse, 3) having a history of gastrointestinal surgery, 4) having suffered from serious cardiovascular, neurological, psychiatric, renal or respiratory diseases, 5) having experienced acid regurgitation, heart burn or upper abdominal pain as the predominant symptom, 6) having had gastric atrophy or erosive gastroduodenal lesions and 7) having had cholecystitis, gall-stones or esophagitis. All of the patients were restricted to take medications (such as prokinetic and antinausea drugs) at least 24 hours before scanning. The subject recruitment criteria and evaluation of the symptoms were similar to that in our previous study [3], [19].

### Healthy Controls

Thirty age and gender matched healthy controls were recruited. They had no history of neurological or psychiatric disorders and had refrained from alcohol or drug consumption in the previous one week, and underwent similar basic evaluations including a review of their medical history, a physical examination, electrocardiogram, upper abdominal ultrasound and gastrointestinal endoscopy.

### Behavioral Measures

The Zung Self-Rating Depression Scale (SDS) [20] and the Zung Self-Rating Anxiety Scale (SAS) [21] were applied to quantify the depression/anxiety related symptoms in the present study. Both of the SDS and SAS consisted of 20 items, each of which was scored from 1 to 4. The final scores of the SDS and SAS were calculated by multiplying the raw score by a factor of 1.25. A score less than 53 (SDS) or 50 (SAS) was considered to be in the normal range according to the Chinese norm [22], [23].

The Nepean Dyspepsia Index (NDI) was used for measuring both the symptom severity and the quality of life (QOL) [24]. The translated version of the NDI was certified to be reliable and valid in evaluating the symptom severity and QOL of Chinese FD patients [25]. The symptom index of the NDI was based on 15 dyspepsia-related physical signs. The number 0 represented no symptoms, and higher numbers paralleled worsening of the symptoms. Meanwhile, the QOL index of the NDI included four domains: interference, know/control, eat/drink and sleep/disturb [26].

### Imaging Data Acquisition

A total of 5-minute resting-state functional imaging data were obtained from each subject. Images were collected using a 3T Siemens scanner (Allegra, Siemens Medical System, Erlangen, Germany) at the Huaxi MR Research Center, West China Hospital of Sichuan University, Chengdu, China. In order to minimize the head motion to diminish scanner noise, a standard birdcage head coil was used along with a restraining foam pad. Functional images were acquired with a single-shot gradient–recalled echo planar imaging sequence. (TR/TE: 2000 ms/30 ms, field of view: 240 mm×240 mm, matrix size: 64×64, flip angle: 90°, in-plane resolution: 3.75 mm×3.75 mm, slice thickness: 5 mm thick with no gaps, 30 axial slices). High resolution T1-weighted images were then collected with a volumetric three-dimensional spoiled gradient recall sequence (TR/TE: 1900 ms/2.26 ms, field of view: 240 mm×240 mm, matrix size: 240× 240, flip angle = 9°, in-plane resolution: 1 mm×1 mm, slice thickness = 1 mm, 176 sagittal slices). During the entire session, subjects were instructed to keep eyes closed, not to think about anything and to stay awake.

### Imaging Data Preprocessing and ReHo Processing

Preprocessing of the resting-state fMRI data were conducted using SPM5 (http://www.fil.ion.ucl.ac.uk/spm/software/spm5). The first 10 volumes of each functional time series were discarded to allow the longitudinal magnetization to reach a steady state and to allow participants acclimate to the scanning environment. The remaining images were corrected for acquisition time delay between different slices and realigned to the first volume. Head motion parameters were computed by estimating translation in each direction and angular rotation on each axis for each volume, which provided a record of head position. We controlled for head motion using a threshold of 1.5 mm translation in any cardinal direction and 1.5 rotation in each of the orthogonal x, y and z axes. The realigned functional volumes were then spatially normalized to the MNI space using the normalization parameters estimated by T1 structural image unified segmentation, re-sampled to 3 mm^3^ voxel. To conserve the fine-grained local pattern and to avoid artificial connections, normalized data were not smoothed. Several sources of spurious variance, including estimated motion parameters, linear drift, and average BOLD signals in ventricular and white matter regions, were removed from the data through linear regression. After the removal of variance, a temporal filtration (0.01–0.08 Hz) was performed to reduce the effect of low-frequency drifts and high-frequency noise. Kendall’s coefficient of concordance (KCC) was used to measure the correlation of the time series of a given voxel with the time series of its 26 nearest neighbors. Individual ReHo maps were generated by calculating KCC within a gray matter mask in a voxel-wise manner using REST software (http://restfmri.net/forum/index.php). When the center cube was on the edge of the gray matter mask, we only calculated ReHo for a voxel if all of remaining nearest voxels were within the gray matter mask. For each subject, KCC map was normalized by dividing KCC in each voxel by the mean KCC of total gray matter.

### Pattern Classification

**Figure 1:**
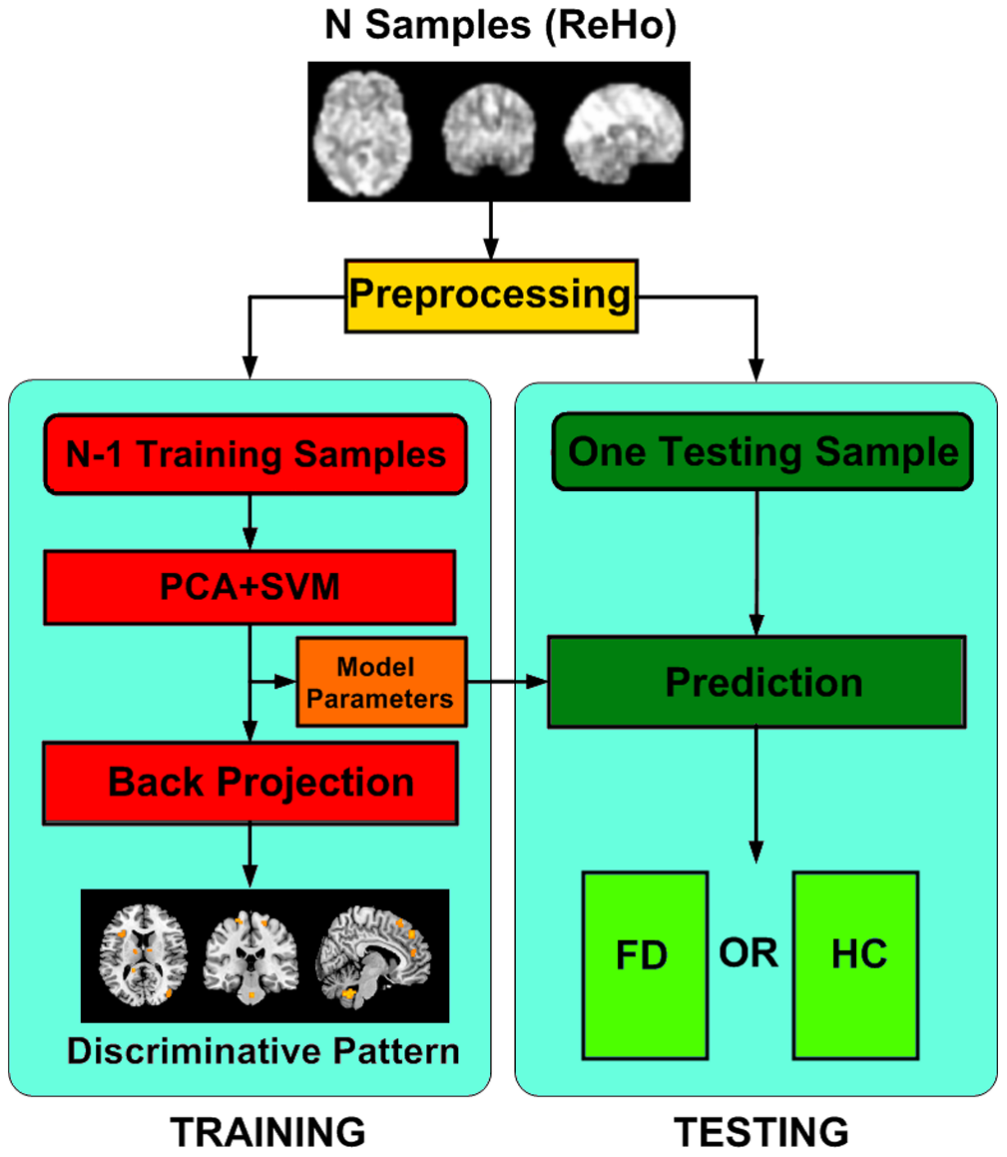
Flowchart outline of the analysis stream of the MVPA method.

The flowchart outline of the analysis stream of the MVPA method was shown in Figure 1. There were two classes of our samples, each with a feature vector x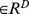 that was extracted from the ReHo images. In the present study, we could obtain a data set N
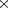
D, where N was the number of training samples, and D was the number of brain voxels in the ReHo map. Since N was far smaller than the feature vector dimensionality D, the computation of the classifier would be complicated and current computers might be even unable to do it. Thereby, it was essential to reduce the dimension. Here, we used principal component analysis (PCA) to project samples into a space of smaller dimensionality without losing information [27]. This approach had been previously applied to reduce the dimensions of the feature space in the neuroimaging field [28], [29]. After the application of PCA to the feature vectors, a set of principle components that explained the variance in the original features were obtained so the dimensionality of the original feature space could be substantially reduced. The low-dimension vector in the new feature space could be acquired by 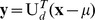, (suppose y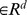). Here, y corresponded to the low-dimension feature vector, 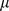 to the mean feature vector of x. PCA would first find the eigenvectors and eigenvalues of the covariance matrix of the feature vector x. Since D far exceeded N, there would be only N–1 meaning eigenvectors according to the linear algebra. That was, the corresponding eigenvalues associated with the N–1 principle components were nonzero and the other remaining eigenvalues are zero. So 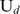
(d = N–1) was a matrix that contained the corresponding eigenvectors of the covariance matrix. In this study, we used all of the principle components so the PCA was a nondestructive dimension reduction and a change in the coordinate system to the subspace.

After dimensionality reduction, support vector machine (SVM) was adopted as the classification algorithm that attempted to find a linear optimal separating hyperplane [11]. This optimal hyperplane with the best generalization was capable of separating the patterns of two classes (FD patients and HCs). The hyperplane was also called the decision boundary that might map fMRI features to brain states. If the training data was not separable, kernel trick and slack variables could be used to solve the problem. By using a kernel trick, the SVM could map the original features into a higher dimension space and attempt to find a linear optimal separating Hyperplane in this high-dimensional space.

The patterns were extracted from ReHo images and the known class label for each sample was fed into a linear kernel support vector machine classifier (LIBSVM–http://www.csie.ntu.edu.tw/~cjlin/libsvm). A hyperplane in the feature space 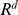 was then enrolled to separate the two classes using the decision function 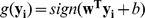, here 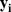 was the pattern of sample i, 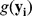 corresponded to the class label for a given pattern 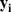,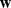 was a normal weight vector to the separating hyperplane and b was an offset. The weight vector 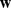
could be treated as an image (i.e. discriminative map) that contained the value of the weight in each voxel. In addition, the parameter C (C = 1, default value [9], [30]) that determined the trade-off between classifier complexity and the number of inseparable patterns was fixed for all situations [10].

We applied the LOOCV test to evaluate the performance of the classifier in separating FD patients and HCs. In each train, data from all but one subject (N–1 subjects) were used to train the classifier, and the classifier was then tested by using the data of the remaining subject. The procedure was repeated N times until each subject was left out once. Finally, Generalization rate (GR), Sensitivity (SS), and Specificity (SC) were calculated to quantify the results of this classifier.
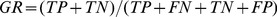
(1)

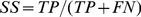
(2)

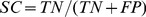
(3)


Where TP is the number of the class label of patients that was correctly predicted; TN is the number of the class label of healthy controls that was correctly predicted; FP is the number of healthy controls classified as patients; and FN is the number of patient classified as healthy controls. So, we defined GR, SS and SC as the overall proportion of subjects that was correctly predicted, the proportion of patients that was correctly predicted, and the proportion of healthy controls that was correctly predicted respectively.

### Discriminative Pattern

Previous studies have shown that the direction of the weight vector is along which the features of two different categories differ most [31], [32]. That means that the value of the weight of one voxel determines its importance in the separation of two brain states. So, we chose the weight vector as the discriminating volume. As determined from the results mentioned above, **w** was obtained in the low-dimension space so it was impossible to directly determine the most discriminating areas. In order to get the discriminative map, we had to map the weight vector **w** back to the original space (i.e. voxel space) by the function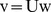, where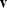 represents the weights of all voxels in the ReHo map.

### Permutation Test

In order to identify the most important brain regions for discriminating between FD and healthy controls, we performed a permutation test to assess the significance. In this study, the value of each voxel in the discrimination map was the object of the statistics. To generate the probability distribution by a permutation test, the class labels were permuted 5,000 times. In each permutation, a linear SVM classifier was trained with the permutation of the labels. A probability map that contained the p value of each voxel in the discrimination map was then derived under the null hypothesis that there was no difference in ReHo maps between FD patients and healthy controls. These voxels in which the p value was under the threshold 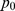 were defined as significant regions. Considering the risk of Type I errors and Type II errors happening, a cluster level testing was performed (1999-Global, Voxel, and Cluster Tests, by Theory and Permutation, for a Difference Between Two Groups of Structural MR Images of the Brain). In order to obtain the clusters (cluster size was set to 10 voxels), the discriminative map at voxel level was first thresholded at 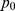 = 0.02. Then we identified the significant clusters between FD patients and healthy controls at the 0.001 level in the nonparametric distribution [33].

### Correlation Analysis

Certain ROIs were defined from the results of MVPA mentioned above. The center Montréal Neurological Institute (MNI) coordinates of the 6 mm sphere was on the base of the largest absolute value of classification weight in each of the significant clusters in the ReHo image. To identify potential interactions between the ROIs in FD patients and the disease symptoms, the mean ReHo value of the voxels in the ROIs of each patient was extracted and correlated with FD duration and/or symptom severity using Pearson’s correlation analysis. Adjustment for multiple comparisons was made with the Bonferroni correction (p<0.01).

## Results

### Behavioral Data Result

The demographics and clinical data of the subjects, between FD patients and HCs, were shown in Table 1. The mean NDI scores were 48.73±17.04 in the FD group, and the mean SDS and SAS scores reported by patients were 43.04±10.20 and 41.79±8.49 respectively. All of these behavioral scores were larger than the ones for HCs. Meanwhile, significant statistical differences were not found in the demographics related to age and sex.

**Table 1:** Behavioral data between FD patients and healthy controls.

Variable	Mean±SD			P-value
	FD patient		Healthy control	
**Age (years)**	22.50±1.46	22.23±0.94		0.40^b^
**Gender(Female/Male)**	20/10	19/11		0.79^a^
**NDI**	48.73±17.04	1.37±2.51		<0.001^b^
**SDS**	43.04±10.20	33.38±6.62		<0.001^b^
**SAS**	41.79±8.49	33.83±6.29		<0.001^b^
**DD (months)**	35.77±22.44			

**Abbreviation:** FD: Functional Dyspepsia; SD: Standard Deviation; NDI: Nepean Dyspepsia Index; SDS: Zung Self-Rating Depression Scale; SAS: Zung Self-Rating Anxiety Scale; DD: Duration of disease.

a The p-value was obtained by Chi-square.

b The p-value was obtained by two-sample two-tailed t-test.

### MVPA Result

There were 60 samples for discriminative analysis, including 30 FD patients and 30 HCs. The classifier was trained with the 60 samples and was tested by using the same 60 samples. That meant a 60-round LOOCV was done to estimate the classifier’s ability of prediction. The GR stemming from ReHo image was about 86.67%, with SS of 83.33% and SC of 90.00%.

**Figure 2:**
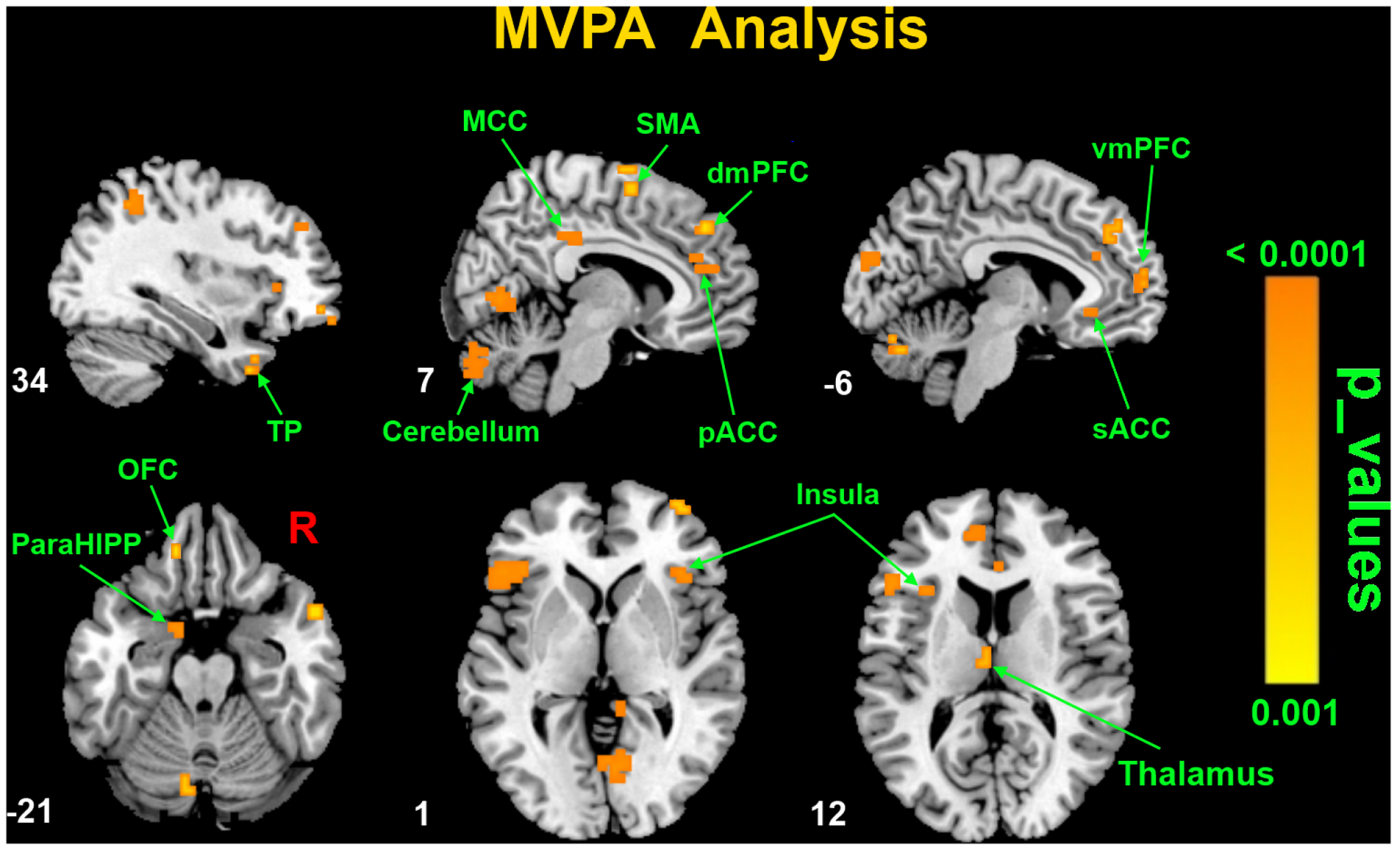
Spatial maps of classifier accuracies for distinguishing a ReHo map between FD patients and healthy controls using the MVPA analysis. Color bar indicates the classification accuracy of the detected brain regions.

The spatial patterns, showing the differences between FD patients and HCs, were displayed in Figure 2 and Table 2 (permutation test: voxel level p<0.02; cluster level p<0.001). The discriminative regions included the right dorsomedial prefrontal cortex (dmPFC), left ventromedial prefrontal cortex (vmPFC), left orbitofrontal cortex (OFC), right supplementary motor area (SMA), right temporal pole (TP), bilateral insula, right pregenual ACC (pACC), left subgenual ACC (sACC), right MCC, left thalamus, left HIPP/ParaHIPP and right cerebellum.

**Table 2:** The MVPA results showing the discriminative pattern between FD patients and healthy controls.

Regions	Hem	BA	MNI			P-Value (voxel-wise)	P-value (cluster-level)
			X	Y	Z		
dmPFC	L						
	R	9	7	42	39	0.0104	0.0008
vmPFC	L	10	−6	55	15	0.0131	<0.0001
	R						
OFC	L	11	−12	43	−21	0.0078	0.001
	R						
SMA	L						
	R	6	7	2	73	0.0162	0.001
TP	L						
	R	20	34	15	−37	0.0192	0.0008
Insula	L	48	−33	20	12	0.0118	0.0004
	R	47	39	24	1	0.0052	0.0002
pACC	L						
	R	32	7	38	24	0.0126	<0.0001
sACC	L	11	−6	36	−7	0.0156	<0.0001
	R						
MCC	L						
	R	23/32	7	−30	36	0.0182	<0.0001
Thalamus	L		−3	−12	12	0.0196	0.001
	R						
HIPP/ParaHIPP	L		−13	4	−21	0.0176	0.0002
	R						
Cerebellum	L						
	R		7	−82	−36	0.0096	<0.0001

**Abbreviation:** Hem: hemisphere; BA: Brodmann area; dmPFC: dorsomedial prefrontal cortex; vmPFC: ventromedial prefrontal cortex; OFC: orbitofrontal cortex; SMA: supplementary motor area; TP: temporal pole; pACC: pregenual anterior cingulate cortex; sACC: subgenual anterior cingulate cortex; MCC: middle cingulated cortex; HIPP: hippocampus; ParaHIPP: parahippocamus.

### Correlation Analysis Result

**Figure 3:**
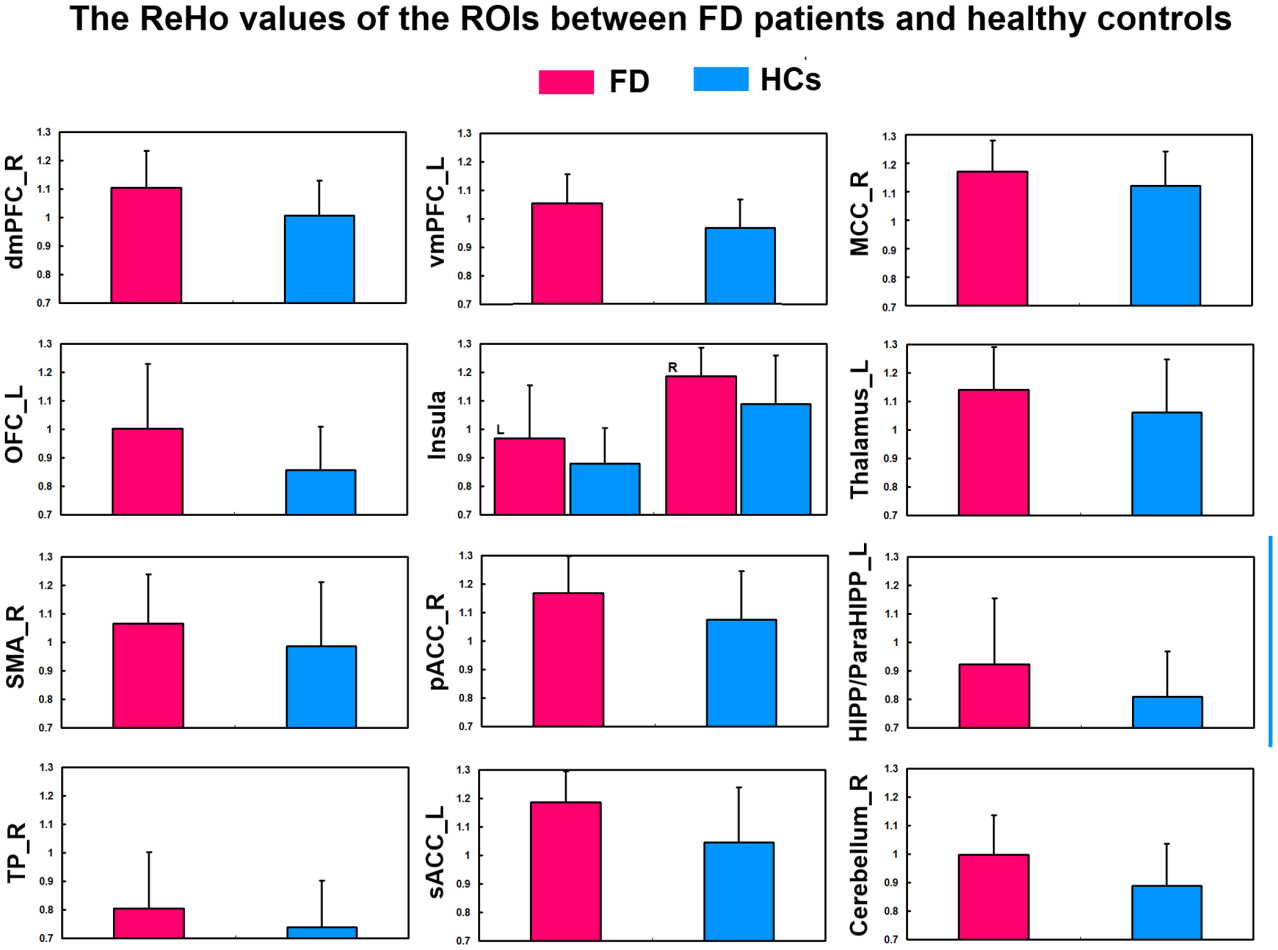
The ReHo values of the ROIs between FD patients and healthy controls.

**Figure 4:**
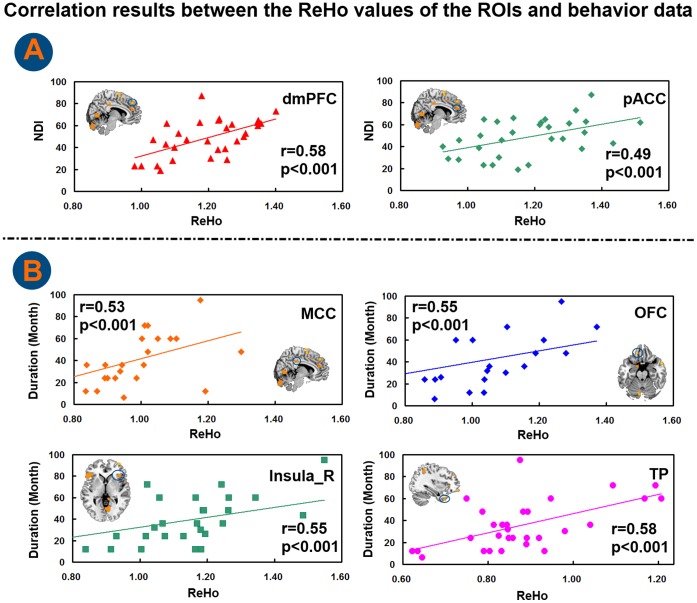
Correlation results between the ReHo values of ROIs and behavior data.

The Figure 3 showed the mean ReHo values of these discriminative regions which were seem as the ROIs. It was shown in Figure 4 that significant correlations were observed between the NDI scores (Figure 4A) and the ReHo values of ROIs and between the FD duration and the ReHo values of ROIs (Figure 4B). In detail, we found significantly positive correlations with the dmPFC (r = 0.58, p<0.001), pACC (r = 0.49, p<0.001) and the FD NDI scores, Meanwhile, we also found significantly positive correlations with the MCC (r = 0.53, p<0.001), OFC (r = 0.55, p<0.001), insula (r = 0.55, p<0.001), TP (r = 0.58, p<0.001) and the disease duration.

## Discussion

The goal of the current study was to increase our understanding of the neural mechanisms of FD patients by both examining the disruption of brain activity in the baseline state and examining interactions between the dysfunctional brain regions and FD symptom. We applied MVPA to identify the distinct neural response patterns between FD patients and HCs. MVPA effectively extracted reliable differences between FD patients’ and HCs’ brains with a generalization rate of 86.67%, sensitivity of 83.33% and specificity of 90.00%. The results revealed that FD-related differences in ReHo were widely found in the brain regions, including the prefrontal cortex, orbitofrontal cortex, supplementary motor area, temporal pole, insula, anterior/middle cingulate cortex, thalamus, hippocampus/parahippocamus and cerebellum. And certain regions were associated with FD symptoms.

Previous studies have detected the ‘gastric sensation neuromatrix’ mainly including the ACC, MCC, insula, PFC, temporal cortex, thalamus and cerebellum [34], [35], [36], [37]. In our present findings, many differences between FD patients and HCs were consistent with these studies. Furthermore, these brain regions were associated with the homeostatic-afferent, emotional-arousal and cortical-modulatory networks, as proposed by Mayer et al [4], [5].

The vmPFC has reciprocal connections with the limbic regions, such as the amygdala and HIPP, and is involved in motor control, monitoring, higher order sensory processing and supporting the regulation of behavior and control of responses to environmental stimuli. Hall et al detected that the activated vmPFC regions suggested affective responses to the distension and cortical modulation of bodily somatic reaction in irritable bowel syndrome (IBS) patients [38]. The engagement of the dmPFC was found during anticipation of aversive stimuli and during tasks of emotional evaluations or integrations [39], [40]. Abnormal changes in both the vmPFC and dmPFC were also found in the default mode network [41]. Considering the higher average ReHo values in the two brain regions, we speculated that FD patients might be more sensitive to the aversive experiments and might have abnormal ability of modulating emotion, which derived from the stomach in the FD patients. The positive correlation between the dmPFC and NDI might presented the degree of dysfunction in the dmPFC might be modulated even in the resting state, following changes in symptom severity.

OFC has a role in discriminating feelings and producing avoidance behaviors. Neuroscience studies show that the OFC is a nexus for sensory integration, monitoring and mapping visceral responses and internal states, as well as appraising sensory reactions and modulating autonomic responses [7], [42]. Moreover, the OFC is related to a variety of functions, especially higher-order executive functions which include control and inhibition of inappropriate behavioral and emotional responses. Since the OFC plays a major role in the homeostatic afferent network, we hypothesized that dysfunctions in the OFC,with the higher average ReHo values, disturbed homeostatic afferent processing in FD patients. The positive correlation further showed the long aversive experiment of FD could make OFC have a worse effect on homeostatic afferent processing.

The insular cortex, as an important brain region, is associated with coordinating visceral sensory and motor information and is involved in autonomic regulation. The insula integrates physiological conditions via homeostatic afferents [10], [13]. The activation in the insula was commonly found in the gastric distension paradigm [3], [43], [44]. Moreover, Philips et al found that anterior insula and dorsal cingulate played an important role in processing and modulating visceral sensation and these regions might belong to a more extensive cortical network involved in integrating emotional and visceral information [45]. The anterior insula is also thought to have an important role in the perception and subjective experience of pain by integrating other brain regions related to sensory and emotional aspects of pain [46]. In this study, it was found that the FD patients had a higher average ReHo in the bilateral insula compared with HCs. And the ReHo in the right insula correlated with FD duration. The present results therefore might show that abnormal insula patterns reflected imbalance of homeostatic input and dysregulation of visceral responses in FD patients, which was driven by FD duration.

CC plays a crucial role in integrating multimodal information important for sensorimotor, emotional, allostatic/homeostatic, and cognitive functions [47], [48], [49]. It has been shown that the pACC, as a subregion of part of the CC, is reciprocally connected to the insular cortex and engages in visceral sensory processing. The pACC could receive a large input from the amygdala and project the information to the visceromotor centers [50]. Electrical stimulation in the pACC could induce autonomic and visceromotor responses [51], [52]. Meanwhile, the pACC is associated with inhibitory pain modulation and cognitive modulation of pain emotion regulation [47]. Another part of CC (sACC) is involved in autonomic and classical conditioning functions, such as sad events [47]. Furthermore, the sACC is implicated in pain inhibition and emotional arousal [53]. Berman et al found deactivation in the sACC deactivation during mild-to-moderate rectal distension in IBS patients [24]. Rignel et al reported reduced activation of the sACC in IBS, particularly with abuse history. It speculated that dysfunctional sACC might be involved in regulation of emotional arousal and indirectly, through its inhibitory effect on amygdala-related circuits, in pain inhibition [54]. On the other hand, the MCC is enrolled in coordinating fear and avoidance of noxious stimuli [55]. Several studies have shown activation of the MCC to noxious somatic and visceral stimulation [56], [57]. Taking into account CC as important hubs of the homeostatic afferent and emotional-arousal networks, our related-CC results might present dysfunctions of pain inhibitory, cognitive and emotional regulation of pain as well as emotional arousal in FD patients, because of the higher ReHo in the CC. Moreover, CC was associated with disease duration and/or symptom severity (in Figure 4). It might present that CC was more a characteristic brain region in FD patients. The interaction between an abnormal CC and the neural mechanism of FD should be further evaluated in future studies.

The different pattern related to the thalamus was found in the present study. The thalamus is a kind of switchboard of information with multiple functions. Furthermore, it is generally believed to act as a relay between a variety of subcortical areas and the cerebral cortex. It relays sensory and motor signals to the cortex [43]. It has been shown that the visceral sensory pathway is to the thalamus, and has an ability of composing sympathetic and parasympathetic afferents [43]. Thereby, the different pattern in the thalamus would be likely to suggest dysfunctions of sensory pathway. Because thalamus is an important part of homeostatic afferent processing network, the thalamus-related dysfunctions might bias FD patients toward unpleasant or painful perceptions from the viscera.

The TP which is located at the anterior-most part of the temporal lobe receives and sends connections to the amygdala and OFC, two regions that have been most commonly linked to socioemotional processing. Thus, it has often been known as a paralimbic region. In addition, the TP and hypothalamus, an important region for autonomic regulation of emotions, have been shown to have a strong connection [58]. TP dysfunction has been associated with numerous socioemotional disorders [35], [58]. In our results, we found the discriminated pattern in the TP in FD patients, compared to HCs. We suggested that the TP was enrolled in the neuropsychological mechanism of emotion in FD patients, which was correlated with disease duration.

As a critical role, HIPP/ParaHIPP is commonly connected with the processing of different aspects of pain and visceral sensations, recall of past visceral pain memories and memories of unpleasant visceral sensations, vigilance towards expected pain, autonomic responses and antinociception [59], [60], [61]. In accordance with these studies, our findings could be suggested to enhance negative expectations or sensations in the FD patients.

Although the cerebellum’s fundamental function remains a mystery, many findings suggest that the cerebellum plays a role in multiple functional domains: cognitive, affective, sensory and motor. Lu et al reported that cerebellum was activated by gastric pain induced by fundus distension [35]. Ness et al found that cerebellum was associated with visceral nociceptive processing [15]. It suggested that the cerebellum might indirectly mediate the perception sensation by integrating sensorimotor circuitry. Thereby, we hypothesized that the differences in the cerebellum with the higher ReHo value, showed cerebellar dysfunction in FD patients.

In addition, resting-state fMRI, ever since its first reported by Biswal et al [62], has been being paid more attention to, and the level of spontaneous low-frequency (0.01–0.01 Hz) fluctuations of blood oxygenation level-dependent (BOLD) signals has become a powerful tool in the investigation of brain disorders [63]. Unlike other related-resting analysis approaches measure the signal synchrony of low-frequency fluctuation activity among different brain areas, ReHo premises that within a functional cluster, the hemodynamic characteristics of every voxel should be similar, or synchronous with that of each other; and the similarity could be modulated by different experiment conditions [63]. It thereby provides more information of regional spontaneous activity [64]. ReHo has been extensively used in studying spontaneous activities from healthy subjects to patients with certain diseases [64], [65], and ReHo mapping was also applied for MVPA [16], [33]. MVPA on resting-state functional MRI data has a better ability of indicating potential neuroimaging-based biomarkers to differentiate patients from HCs at the individual subject level and potentially finding spatially distributed information to highlight the neural mechanisms underlying the differentiate symptoms of certain disorders. In the present study, the MVPA results showed a wider range of brain regions which could distinguish FD patients from HCs in terms of resting-state functional MRI. The present results suggested that a suitable MVPA approach was able to identify both pattern discrimination and pattern localization during the resting-state, associated with FD. All of our findings reflected a significant methodological contribution in the brain-gut interaction research.

### Limitations

The limitations in the present study should be noted. Firstly, the FD patients were not divided into hyper- and normosensitive groups. We will try to investigate the different patterns among hyper-, normosensitive FD patients and HCs in a future study. Secondly, how to combine functional imaging and other neuroimaging, such as structural magnetic resonance imaging and/or diffusion tensor imaging, will be also investigated in the future.

### Conclusions

To summarize, the present study demonstrated that the different patterns in the brain between FD patients and HCs could be decoded by using an SVM-based MVPA approach, combined with a ReHo map. Here, the discrepancies in activation patterns were mainly characterized in the PFC, OFC, SMA, TP, insula, CC, thalamus, HIPP/ParaHIPP and cerebellum. These results verified our hypothesis about FD-related abnormalities of brain activity during the resting state, and provided evidence that MVPA approach was effective in analyzing them. It was hoped that the current study could provided useful information for the mechanism underlying FD and related diagnostic assay.

## References

[1] Tack J, Talley NJ, Camilleri M, Holtmann G, Hu P, et al (2006) Functional gastroduodenal disorders. Gastroenterology 130: 1466–1479. 16678560 10.1053/j.gastro.2005.11.059

[2] Van Oudenhove L, Vandenberghe J, Dupont P, Geeraerts B, Vos R, et al (2010) Abnormal regional brain activity during rest and (anticipated) gastric distension in functional dyspepsia and the role of anxiety: A H_2_ ^15^O -PET study. Am J Gastroenterol 105: 913–924. 20160711 10.1038/ajg.2010.39

[3] Zeng F, Qin W, Liang F, Liu J, Tang Y, et al (2011) Abnormal Resting Brain Activity in Patients With Functional Dyspepsia Is Related to Symptom Severity. Gastroenterology 141: 499–506. 21684280 10.1053/j.gastro.2011.05.003

[4] Mayer EA (2011) Gut feelings: the emerging biology of gut–brain communication. Nat Rev Neurosci 12: 453–466. 21750565 10.1038/nrn3071 PMC3845678

[5] Mayer EA, Naliboff BD, Craig A (2006) Neuroimaging of the brain-gut axis: from basic understanding to treatment of functional GI disorders. Gastroenterology 131: 1925–1942. 17188960 10.1053/j.gastro.2006.10.026

[6] Van Oudenhove L, Vandenberghe J, Dupont P, Geeraerts B, Vos R, et al (2010) Regional Brain Activity in Functional Dyspepsia: A H_2_ ^15^O study on the role of gastric sensitivity and abuse history. Gastroenterology 139: 36–47. 20406641 10.1053/j.gastro.2010.04.015

[7] Vandenberghe J, Dupont P, Van Oudenhove L, Bormans G, Demyttenaere K, et al (2007) Regional cerebral blood flow during gastric balloon distention in functional dyspepsia. Gastroenterology 132: 1684–1693. 17484866 10.1053/j.gastro.2007.03.037

[8] Zhou G, Liu P, Zeng F, Yuan K, Yu D, et al.. (2012) Increased interhemispheric resting-state functional connectivity in functional dyspepsia: a pilot study. NMR Biomed. doi: 10.1002/nbm.2878. 10.1002/nbm.2878 23225275

[9] Mourão-Miranda J, Reynaud E, McGlone F, Calvert G, Brammer M (2006) The impact of temporal compression and space selection on SVM analysis of single-subject and multi-subject fMRI data. Neuroimage 33: 1055–1065. 17010645 10.1016/j.neuroimage.2006.08.016

[10] Weygandt M, Schaefer A, Schienle A, Haynes JD (2011) Diagnosing different binge-eating disorders based on reward-related brain activation patterns. Hum Brain Mapp 33: 2135–2146. 22887826 10.1002/hbm.21345 PMC6869845

[11] Pereira F, Mitchell T, Botvinick M (2009) Machine learning classifiers and fMRI: a tutorial overview. Neuroimage 45: S199–209. 19070668 10.1016/j.neuroimage.2008.11.007 PMC2892746

[12] Dosenbach NU, Nardos B, Cohen AL, Fair DA, Power JD, et al (2010) Prediction of individual brain maturity using fMRI. Science 329: 1358–1361. 20829489 10.1126/science.1194144 PMC3135376

[13] Fan Y, Batmanghelich N, Clark CM, Davatzikos C (2008) Spatial patterns of brain atrophy in MCI patients, identified via high-dimensional pattern classification, predict subsequent cognitive decline. Neuroimage 39: 1731–1743. 18053747 10.1016/j.neuroimage.2007.10.031 PMC2861339

[14] Ecker C, Rocha-Rego V, Johnston P, Mourao-Miranda J, Marquand A, et al (2010) Investigating the predictive value of whole-brain structural MR scans in autism: a pattern classification approach. Neuroimage 49: 44–56. 19683584 10.1016/j.neuroimage.2009.08.024

[15] Weygandt M, Blecker CR, Schäfer A, Hackmack K, Haynes JD, et al (2012) fMRI pattern recognition in obsessive–compulsive disorder. Neuroimage 60: 1186–1193. 22281674 10.1016/j.neuroimage.2012.01.064

[16] Zhu CZ, Zang YF, Cao QJ, Yan CG, He Y, et al (2008) Fisher discriminative analysis of resting-state brain function for attention-deficit/hyperactivity disorder. Neuroimage 40: 110–120. 18191584 10.1016/j.neuroimage.2007.11.029

[17] Craddock RC, Holtzheimer PE, Hu XP, Mayberg HS (2009) Disease state prediction from resting state functional connectivity. Magn Reson Med 62: 1619–1628. 19859933 10.1002/mrm.22159 PMC3749911

[18] Zeng LL, Shen H, Liu L, Wang L, Li B, et al (2012) Identifying major depression using whole-brain functional connectivity: a multivariate pattern analysis. Brain 135: 1498–1507. 22418737 10.1093/brain/aws059

[19] Zhou G, Qin W, Zeng F, Liu P, Yang X, et al (2013) White-Matter Microstructural Changes in Functional Dyspepsia: A Diffusion Tensor Imaging Study. Am J Gastroenterol 108: 260–269. 23229422 10.1038/ajg.2012.405

[20] Zung WWK, Richards CB, Short MJ (1965) Self-rating depression scale in an outpatient clinic: further validation of the SDS. Arch Gen Psychiatry 13: 508–515. 4378854 10.1001/archpsyc.1965.01730060026004

[21] Zung WW (1971) A rating instrument for anxiety disorders. Psychosomatics: Journal of consultation liaison psychiatry 12: 371–379. 10.1016/S0033-3182(71)71479-0 5172928

[22] Wenyuan W (1990) Self-Rateing Anxiety Scale (SAS). Shanghai Arch Psychol 2(supplement: on Psychological Rating Scale): 44.

[23] Chunfang W, Zehuan C, Qing X (1986) Self-Rating Depression Scale (SDS): an analysis on 1340 health subjects. Chin J Nerv Men Dis 12: 267–268.

[24] Talley N, Haque M, Wyeth J, Stace N, Tytgat G, et al (1999) Development of a new dyspepsia impact scale: the Nepean Dyspepsia Index. Aliment Pharmacol Ther 13: 225–236. 10102954 10.1046/j.1365-2036.1999.00445.x

[25] Tian XP, Li Y, Liang FR, Sun GJ, Yan J, et al (2009) Translation and validation of the Nepean Dyspepsia Index for functional dyspepsia in China. World J Gastroenterol 15: 3173–3177. 19575499 10.3748/wjg.15.3173 PMC2705742

[26] Talley NJ, Verlinden M, Jones M (1999) Validity of a new quality of life scale for functional dyspepsia: a United States multicenter trial of the Nepean Dyspepsia Index. Am J Gastroenterol 94: 2390–2397. 10483997 10.1111/j.1572-0241.1999.01363.x

[27] Ringnér M (2008) What is principal component analysis? Nat Biotechnol 26: 303–304. 18327243 10.1038/nbt0308-303

[28] Song S, Zhan Z, Long Z, Zhang J, Yao L (2011) Comparative study of SVM methods combined with voxel selection for object category classification on fMRI data. PLoS One 6: e17191. 21359184 10.1371/journal.pone.0017191 PMC3040226

[29] You X, Adjouadi M, Wang J, Guillen MR, Bernal B, et al.. (2012) A decisional space for fMRI pattern separation using the principal component analysis-A comparative study of language networks in pediatric epilepsy. Hum Brain Mapp doi: 10.1002/hbm.22069. 10.1002/hbm.22069 PMC3398237 22461299

[30] Momennejad I, Haynes J-D (2012) Human anterior prefrontal cortex encodes the ‘what’ and ‘when’of future intentions. Neuroimage 61: 139–148. 22418393 10.1016/j.neuroimage.2012.02.079

[31] Mourão-Miranda J, Bokde ALW, Born C, Hampel H, Stetter M (2005) Classifying brain states and determining the discriminating activation patterns: support vector machine on functional MRI data. Neuroimage 28: 980–995. 16275139 10.1016/j.neuroimage.2005.06.070

[32] Mourão-Miranda J, Friston KJ, Brammer M (2007) Dynamic discrimination analysis: a spatial-temporal SVM. Neuroimage 36: 88–99. 17400479 10.1016/j.neuroimage.2007.02.020

[33] Wang L, Shen H, Tang F, Zang Y, Hu D (2012) Combined structural and resting-state functional MRI analysis of sexual dimorphism in the young adult human brain: An MVPA approach. Neuroimage 61: 931–940. 22498657 10.1016/j.neuroimage.2012.03.080

[34] Ladabaum U, Minoshima S, Hasler WL, Cross D, Chey WD, et al (2001) Gastric distention correlates with activation of multiple cortical and subcortical regions. Gastroenterology 120: 369–376. 11159877 10.1053/gast.2001.21201

[35] Lu CL, Wu YT, Yeh TC, Chen LF, Chang FY, et al (2004) Neuronal correlates of gastric pain induced by fundus distension: a 3T-fMRI study. Neurogastroenterol Motil 16: 575–587. 15500514 10.1111/j.1365-2982.2004.00562.x

[36] Vandenbergh J, DuPont P, Fischler B, Bormans G, Persoons P, et al (2005) Regional brain activation during proximal stomach distention in humans: a positron emission tomography study. Gastroenterology 128: 564–573. 15765391 10.1053/j.gastro.2004.11.054

[37] Stephan E, Pardo JV, Faris PL, Hartman BK, Kim SW, et al (2003) Functional neuroimaging of gastric distention. J Gastrointest Surg 7: 740–749. 13129550 10.1016/s1091-255x(03)00071-4

[38] Hall G, Kamath M, Collins S, Ganguli S, Spaziani R, et al (2010) Heightened central affective response to visceral sensations of pain and discomfort in IBS. Neurogastroenterol Motil 22: 276–280. 20003075 10.1111/j.1365-2982.2009.01436.x

[39] Ploghaus A, Tracey I, Gati JS, Clare S, Menon RS, et al (1999) Dissociating pain from its anticipation in the human brain. Science 284: 1979–1981. 10373114 10.1126/science.284.5422.1979

[40] Wiech K, Seymour B, Kalisch R, Enno Stephan K, Koltzenburg M, et al (2005) Modulation of pain processing in hyperalgesia by cognitive demand. Neuroimage 27: 59–69. 15978845 10.1016/j.neuroimage.2005.03.044

[41] Liu P, Zeng F, Zhou G, Wang J, Wen H, et al.. (2013) Alterations of the default mode network in functional dyspepsia patients: a resting-state fmri study. Neurogastroenterol Motil. doi: 10.1111/nmo.12131. 10.1111/nmo.12131 23617737

[42] Kringelbach ML (2005) The human orbitofrontal cortex: linking reward to hedonic experience. Nat Rev Neurosci 6: 691–702. 16136173 10.1038/nrn1747

[43] Saper CB (2002) The central autonomic nervous system: conscious visceral perception and autonomic pattern generation. Annu Rev Neurosci 25: 433–469. 12052916 10.1146/annurev.neuro.25.032502.111311

[44] Mayer EA, Craske M, Naliboff BD (2001) Depression, anxiety, and the gastrointestinal system. J Clin Psychiatry 62: 28–36. 12108819

[45] Phillips ML, Gregory LJ, Cullen S, Cohen S, Ng V, et al (2003) The effect of negative emotional context on neural and behavioural responses to oesophageal stimulation. Brain 126: 669–684. 12566287 10.1093/brain/awg065

[46] Berman SM, Naliboff BD, Suyenobu B, Labus JS, Stains J, et al (2008) Reduced brainstem inhibition during anticipated pelvic visceral pain correlates with enhanced brain response to the visceral stimulus in women with irritable bowel syndrome. J Neurosci 28: 349–359. 18184777 10.1523/JNEUROSCI.2500-07.2008 PMC6670525

[47] Vogt BA (2005) Pain and emotion interactions in subregions of the cingulate gyrus. Nat Rev Neurosci 6: 533–544. 15995724 10.1038/nrn1704 PMC2659949

[48] Paus T (2001) Primate anterior cingulate cortex: where motor control, drive and cognition interface. Nat Rev Neurosci 2: 417–424. 11389475 10.1038/35077500

[49] Taylor KS, Seminowicz DA, Davis KD (2008) Two systems of resting state connectivity between the insula and cingulate cortex. Hum Brain Mapp 30: 2731–2745. 10.1002/hbm.20705 PMC6871122 19072897

[50] Clark DL, Boutros NN, Mendez MF (2010) The brain and behavior: an introduction to behavioral neuroanatomy: Cambridge: Cambridge University Press.

[51] Devinsky O, Morrell MJ, Vogt BA (1995) Contributions of anterior cingulate cortex to behaviour. Brain 118: 279–306. 7895011 10.1093/brain/118.1.279

[52] Talairach J, Bancaud J, Geier S, Bordas-Ferrer M, Bonis A, et al (1973) The cingulate gyrus and human behaviour. Electroencephalogr Clinl Neurophysiol 34: 45–52. 10.1016/0013-4694(73)90149-1 4118434

[53] Valet M, Sprenger T, Boecker H, Willoch F, Rummeny E, et al (2004) Distraction modulates connectivity of the cingulo-frontal cortex and the midbrain during pain–an fMRI analysis. Pain 109: 399–408. 15157701 10.1016/j.pain.2004.02.033

[54] Ringel Y, Drossman DA, Leserman JL, Suyenobu BY, Wilber K, et al (2008) Effect of abuse history on pain reports and brain responses to aversive visceral stimulation: an FMRI study. Gastroenterology 134: 396–404. 18242208 10.1053/j.gastro.2007.11.011

[55] Lee HF, Hsieh JC, Lu CL, Yeh TC, Tu CH, et al (2012) Enhanced affect/cognition-related brain responses during visceral placebo analgesia in irritable bowel syndrome patients. Pain 153: 1301–1310. 22541443 10.1016/j.pain.2012.03.018

[56] Elsenbruch S, Rosenberger C, Enck P, Forsting M, Schedlowski M, et al (2010) Affective disturbances modulate the neural processing of visceral pain stimuli in irritable bowel syndrome: an fMRI study. Gut 59: 489–495. 19651629 10.1136/gut.2008.175000

[57] Larsson M, Tillisch K, Craig A, Engström M, Labus J, et al (2012) Brain responses to visceral stimuli reflect visceral sensitivity thresholds in patients with irritable bowel syndrome. Gastroenterology 142: 463–472. 22108191 10.1053/j.gastro.2011.11.022 PMC3288538

[58] Olson IR, Plotzker A, Ezzyat Y (2007) The enigmatic temporal pole: a review of findings on social and emotional processing. Brain 130: 1718–1731. 17392317 10.1093/brain/awm052

[59] Wilder-Smith C, Schindler D, Lovblad K, Redmond S, Nirkko A (2004) Brain functional magnetic resonance imaging of rectal pain and activation of endogenous inhibitory mechanisms in irritable bowel syndrome patient subgroups and healthy controls. Gut 53: 1595–1601. 15479679 10.1136/gut.2003.028514 PMC1774298

[60] Mayer E, Naliboff B, Lee O, Munakata J, Chang L (2001) Review article: gender-related differences in functional gastrointestinal disorders. Aliment Pharmacol Ther 13: 65–69. 10.1046/j.1365-2036.1999.00008.x 10429743

[61] Andresen V, Bach D, Poellinger A, Tsrouya C, Stroh A, et al (2005) Brain activation responses to subliminal or supraliminal rectal stimuli and to auditory stimuli in irritable bowel syndrome. Neurogastroenterol Motil 17: 827–837. 16336498 10.1111/j.1365-2982.2005.00720.x

[62] Biswal B, Yetkin FZ, Haughton VM, Hyde JS (1995) Functional Connectivity in the Motor Cortex of Resting Human Brain Using Echo-Planar MRI. Magn Reson Med 34: 537–537. 8524021 10.1002/mrm.1910340409

[63] Zhang D, Raichle ME (2010) Disease and the brain’s dark energy. Nat Rev Neurol 6: 15–28. 20057496 10.1038/nrneurol.2009.198

[64] Zang Y, Jiang T, Lu Y, He Y, Tian L (2004) Regional homogeneity approach to fMRI data analysis. Neuroimage 22: 394–400. 15110032 10.1016/j.neuroimage.2003.12.030

[65] Song XW, Dong ZY, Long XY, Li SF, Zuo XN, et al (2011) REST: a toolkit for resting-state functional magnetic resonance imaging data processing. PloS one 6: e25031. 21949842 10.1371/journal.pone.0025031 PMC3176805

